# Supplementary material for: PLGA-particle vaccine carrying TLR3/RIG-I ligand Riboxxim synergizes with immune checkpoint blockade for effective anti-cancer immunotherapy
Source: Nat Commun. 2021 May 18;12:2935. doi: 10.1038/s41467-021-23244-3 (PMC8131648; doi:10.1038/s41467-021-23244-3)
Supplement: Supplementary file 1 — Supplementary Information [file 41467_2021_23244_MOESM1_ESM.pdf]

Supplementary information for:

PLGA-particle vaccine carrying TLR3/RIG-I ligand Riboxxim synergizes with immune checkpoint blockade for effective anti-cancer immunotherapy

Julia Koerner[1,#], Dennis Horvath[1,2,#], Valerie L. Herrmann[1,+], Anna MacKerracher[1], Bruno Gander[3], Hideo Yagita[4], Jacques Rohayem[5,6], and Marcus Groettrup*[1,7]

[1]Division of Immunology, Department of Biology, University of Konstanz, Konstanz, Germany

[2]Centre for the Advanced Study of Collective Behaviour, University of Konstanz, Konstanz, Germany

[3]Institute of Pharmaceutical Sciences, ETH Zürich, Zürich, Switzerland

[4]Department of Immunology, Juntendo University School of Medicine, Tokyo, Japan

[5]Riboxx GmbH, BioInnovationszentrum, Dresden, Germany

[6]Institute of Virology, Medical Faculty Carl Gustav Carus, Dresden University of Technology, Dresden, Germany

[7]Biotechnology Institute Thurgau at the University of Konstanz (BITg), Kreuzlingen, Switzerland

[#]These authors contributed equally: Julia Koerner and Dennis Horvath

[+]Present address: Boehringer Ingelheim Pharma, Cancer Immunology + Immune Modulation Biberach/ Riß, Germany

*Corresponding author: Marcus Groettrup, marcus.groettrup@uni-konstanz.de

SUPPLEMENTARY FIGURES

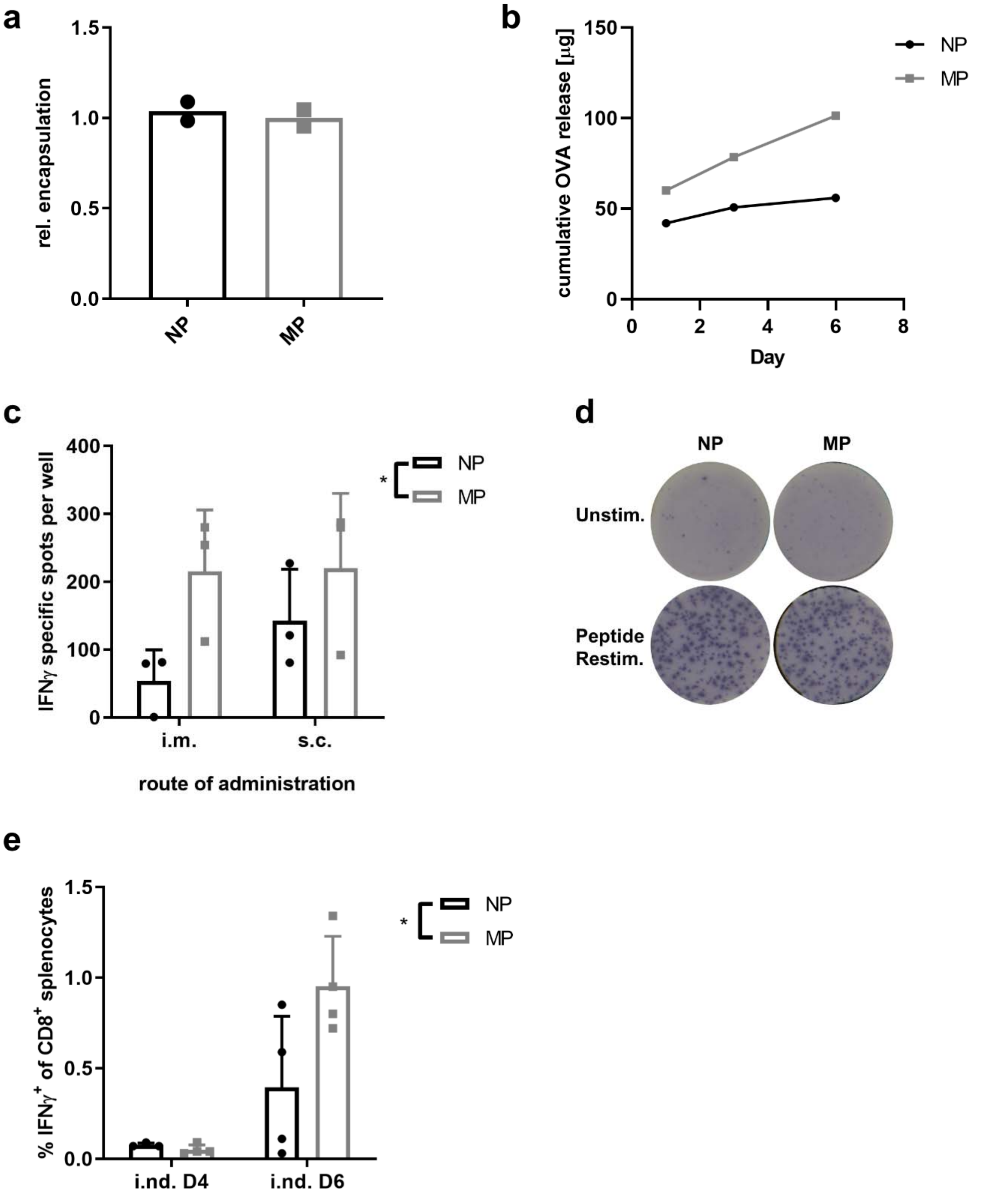

Supplementary Fig. 1 │ Comparison of PLGA nanoparticles (NP) and microparticles (MP) with respect to encapsulation efficiency, release kinetics and T cell priming. a, Relative encapsulation efficiency of OVA into PLGA-NP compared to PLGA-MP. 5 mg of NP (black circles, n=2) or MP-OVA/Riboxxim (grey squares, n=2) (250 µg OVA and 2.5µg Riboxxim)

were dissolved in 50 µl of DMSO. Protein content in the supernatant (SN) was measured by MicroBCA™ and was normalized to the protein content in PLGA-MP. **b**, In vitro release profile of NP and MP-OVA/Riboxxim. 5 mg of NP (black circles, n=2) or MP-OVA/Riboxxim (grey squares, n=2) were incubated at 37°C in 200 µl PBS (pH 7.4). Samples were taken on day 1, 3, and 6 and protein content in the SN was determined by MicroBCA™ assay. The curves present the cumulative release over indicated days, showing slightly increased release profiles for PLGA-MP. **c**, C57BL/6J mice were immunized with 5 mg of either NP (black circles, n=3) or MP-OVA/Riboxxim (grey squares, n=3) (250 µg OVA; 2.5µg Riboxxim) via the intramuscular (i.m.) or subcutaneous (s.c.) route. On day 6 post immunization, an ELISpot assay for IFNγ of splenocytes was performed after ex vivo re-stimulation with SIINFEKL peptide. Statistics: two-way ANOVA with with $^*P < 0.05$. **d**, Representative ELISpot images of IFNγ-positive spot formation of the indicated groups (Peptide Restim.) compared to unstimulated wells (Unstim.), showing similar IFNγ responses between PLGA-NP and PLGA-MP treatments. **e,** C57BL/6J mice were immunized with either NP (black circles, n=4) or MP-OVA/Riboxxim (grey squares, n=4) (0.5 µg OVA/mouse; 0.025 µg Riboxxim/mouse) via intranodal (i.nd.) injection. The efficacy of the cytotoxic T cell response to OVA/Riboxxim particle vaccination was determined on day 4 (D4) and day 6 (D6) after immunization by ELISpot assay for IFNγ. The values in (c) and (d) are given as IFNγ-specific spots per well. Background levels of unstimulated samples were subtracted. Statistical significance was determined by two-way ANOVA with $^*P < 0.05$. All data represent means ± SD.

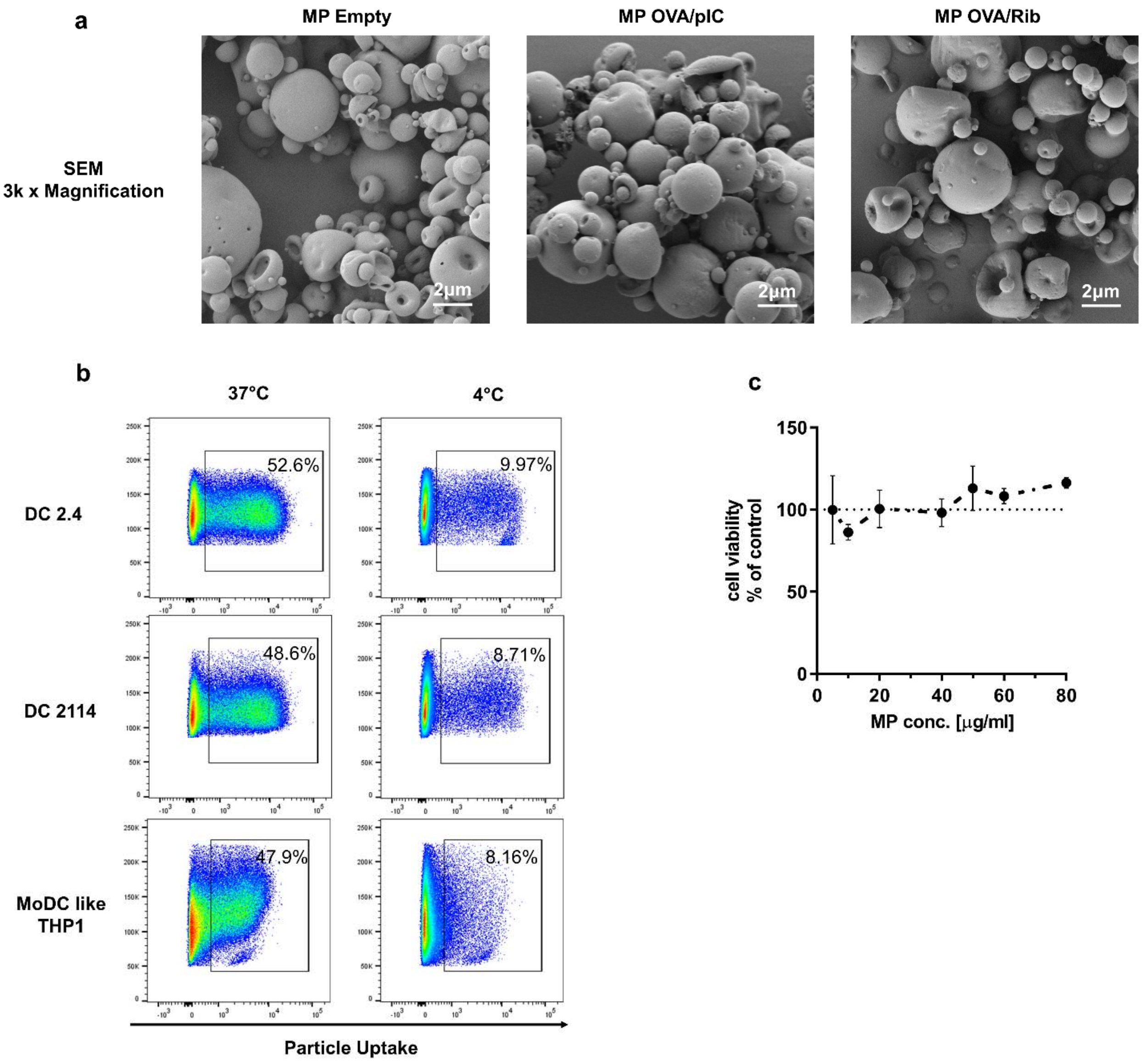

Supplementary Fig. 2 | Physicochemical characterization and uptake of PLGA-MP. **a**, Morphology and particle sizes of PLGA-MP loaded with 250 µg OVA and 2.5 µg dsRNA analogues as indicated above the images were analyzed by scanning electron microscopy (SEM); scale bar: 2 µm, magnification x3000. SEM images were acquired from 3 different particle batches with similar results. Scale bars have been added using ImageJ (FIJI) v1.53c. **b**, Uptake of fluorescently labeled PLGA-MP-QD705/OVA/Riboxxim was analyzed by flow cytometric analysis of QD705 (QuantumDot705) positive signals. Murine DC2.4 and MutuDC2114 cells as well as in vitro differentiated human monocyte-derived dendritic cell

(MoDC)-like THP-1 cells were incubated with MP-QD705/OVA/Riboxxim for 6 h. Incubation at 4°C served as negative control for no-uptake. **c,** Viability of 5x10^4 mouse BMDCs (n=3) after addition of indicated concentrations of PLGA-MP-OVA/Riboxxim at 48 hours. Deep Blue Viability™ assay was performed to determine cell viability compared to control dead cells and untreated cells. Data show means ± SD from three independent experiments with similar outcome.

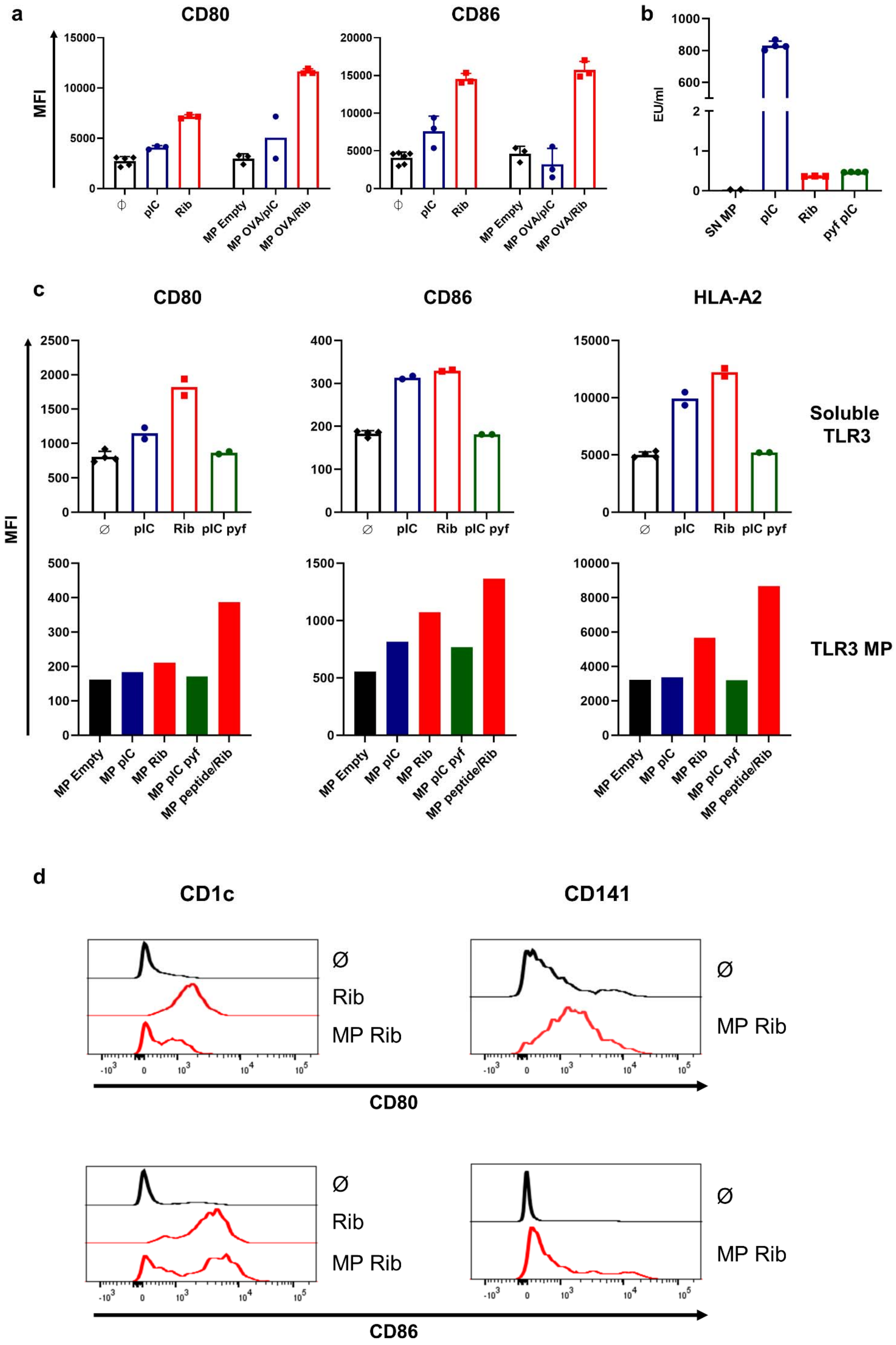
a
CD80
CD86
MFI
15000
10000
5000
0
20000
15000
10000
5000
0
ϕ
pIC
Rib
MP Empty
MP OVA/pIC
MP OVA/Rib
b
EU/ml
1000
800
600
2
1
0
SN MP
pIC
Rib
pyf pIC
c
CD80
CD86
HLA-A2
2500
2000
1500
1000
500
0
400
300
200
100
0
15000
10000
5000
0
∅
pIC
Rib
pIC pyf
Soluble TLR3
500
400
300
200
100
0
1500
1000
500
0
10000
8000
6000
4000
2000
0
MP Empty
MP pIC
MP Rib
MP pIC pyf
MP peptide/Rib
TLR3 MP
d
CD1c
CD141
Ø
Rib
MP Rib
Ø
MP Rib
CD80
Ø
Rib
MP Rib
Ø
MP Rib
CD86

Supplementary Fig. 3 | Activation and maturation of mouse and human dendritic cells in presence of Riboxxim versus polyI:C. a, Surface maturation markers CD80 and CD86 were analyzed by flow cytometric analysis after incubation of mouse BMDCs with 20 µg/mL soluble polyI:C (pIC, blue circles, n=3), Riboxxim (Rib, red squares, n=3) or with 10 µg/mL PLGA-MP loaded with 250 µg OVA protein and 2.5 µg of polyI:C (MP OVA/pIC, blue circles, n=2-3) or Riboxxim (MP OVA/Rib, red squares, n=3) for 48 h. No treatment (Ø, black diamonds, n=5) or incubation with empty MP (MP Empty, black diamonds, n=3) were used as controls. **b**, Endotoxin levels of used dsRNA analogues (polyI:C, pIC, blue circles, n=4; Riboxxim, Rib, red squares, n=3, pyrogen-free polyI:C, pyf pIC, green circles, n=4) as wells as supernatants of empty PLGA particles (SN MP, black diamonds, n=2) dissolved in PBS (pH7.4) for 24 h were determined in vitro by chromogenic LAL Endotoxin Assay. **c,** Cell surface expression of co-stimulatory molecules CD80 and CD86 and of the MHC class I molecule HLA-A2 was analyzed in in vitro differentiated THP-1 monocytoid cells 24 h after stimulation with 20 µg/mL soluble dsRNA adjuvants (upper panels, n=2) or 48 h after incubation with 10µg/ml of indicated PLGA-MP containing only dsRNA analogues (2.5 µg) (n=1) or PLGA-MP carrying 10 mg peptide co-encapsulated with 2.5 µg Riboxxim (MP peptide/Rib, red, n=1) (lower panel), showing improved DC activation in presence of antigen and Riboxxim in PLGA-MP. Mean fluorescence intensity (MFI) data are presented as means ± SD (for $n \geq 3$) from at least 2 independent experiments with similar outcome. **d**, Histogram of DC activation marker CD80 and CD86 of primary peripheral human blood $CD1c^{+}$ and $CD141^{+}$ DCs 24 h after pulsing with 20 µg/mL Riboxxim (Rib, red) or PLGA-MP charged with 2.5 µg Riboxxim (MP Rib, red). Cells left unstimulated (Ø, black) served as control.

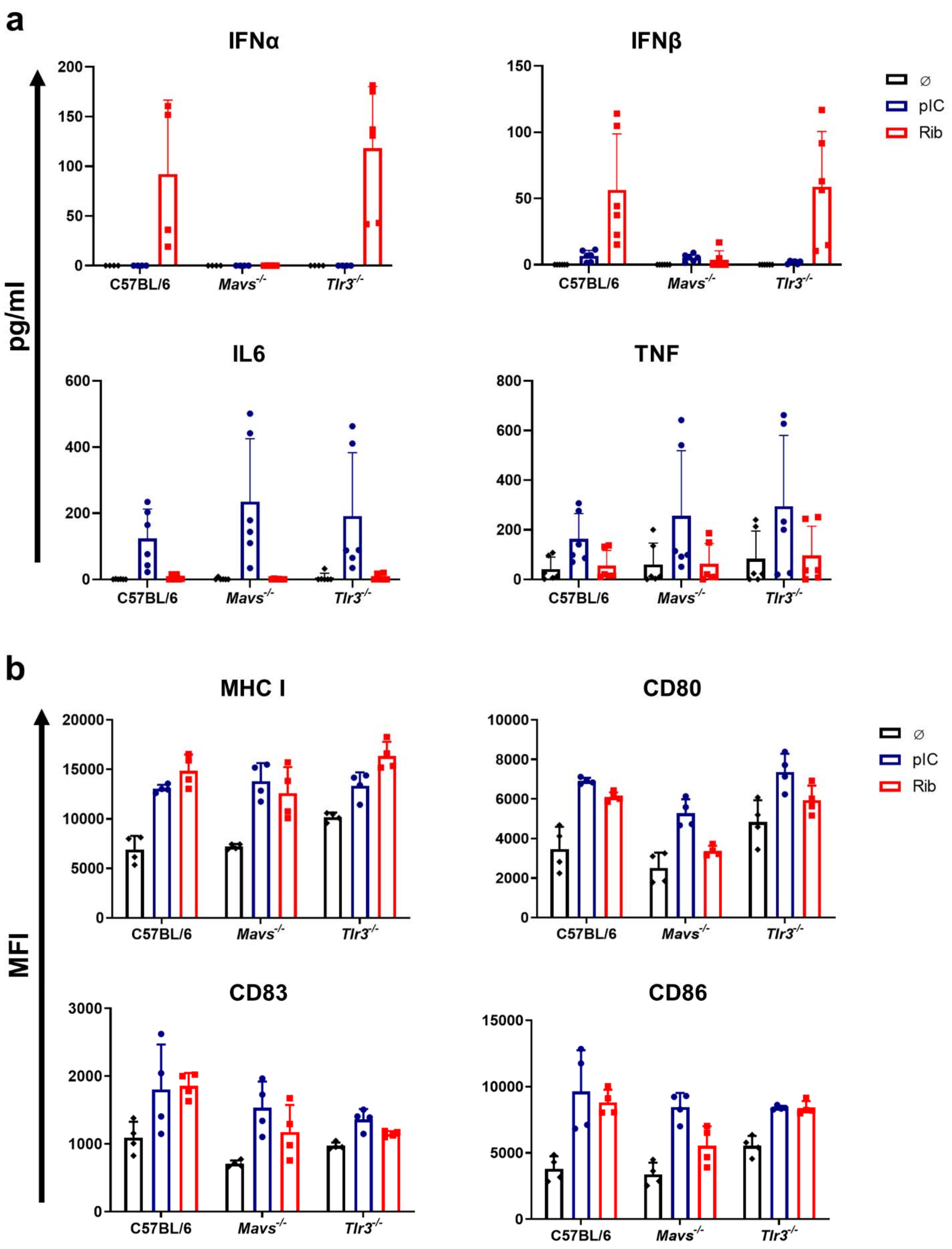
a
IFNα
IFNβ
IL6
TNF
pg/ml
C57BL/6
Mavs-/-
Tlr3-/-
∅
pIC
Rib
b
MHC I
CD80
CD83
CD86
MFI

Supplementary Fig. 4 │ DC maturation and cytokine profile of *Mavs*$^{-/-}$ and *Tlr3*$^{-/-}$ deficient BMDCs after stimulation with Riboxxim or polyI:C. Bone marrow-derived dendritic cells (BMDCs) from wild-type C57BL/6 mice, or mice genetically deficient for *Mavs* or *Tlr3* were stimulated with 20 µg/mL soluble polyI:C (pIC, blue circles) or Riboxxim (Rib, red squares) for 24 h. **a**, Levels of IFNα (n=4), IFNβ (n=6), IL-6 (n=6) and TNF (n=6) in BMDC culture supernatants were determined by ELISA. **b**, Mean fluorescence intensity (MFI) of surface maturation markers CD80 (n=4) and CD86 (n=4), co-stimulatory molecule CD83 (n=4) and MHC class I (H2-K^{b}, n=4) were analyzed by flow cytometry. Data are presented as means ± SD of pooled data from 2 independent experiments with similar outcome.

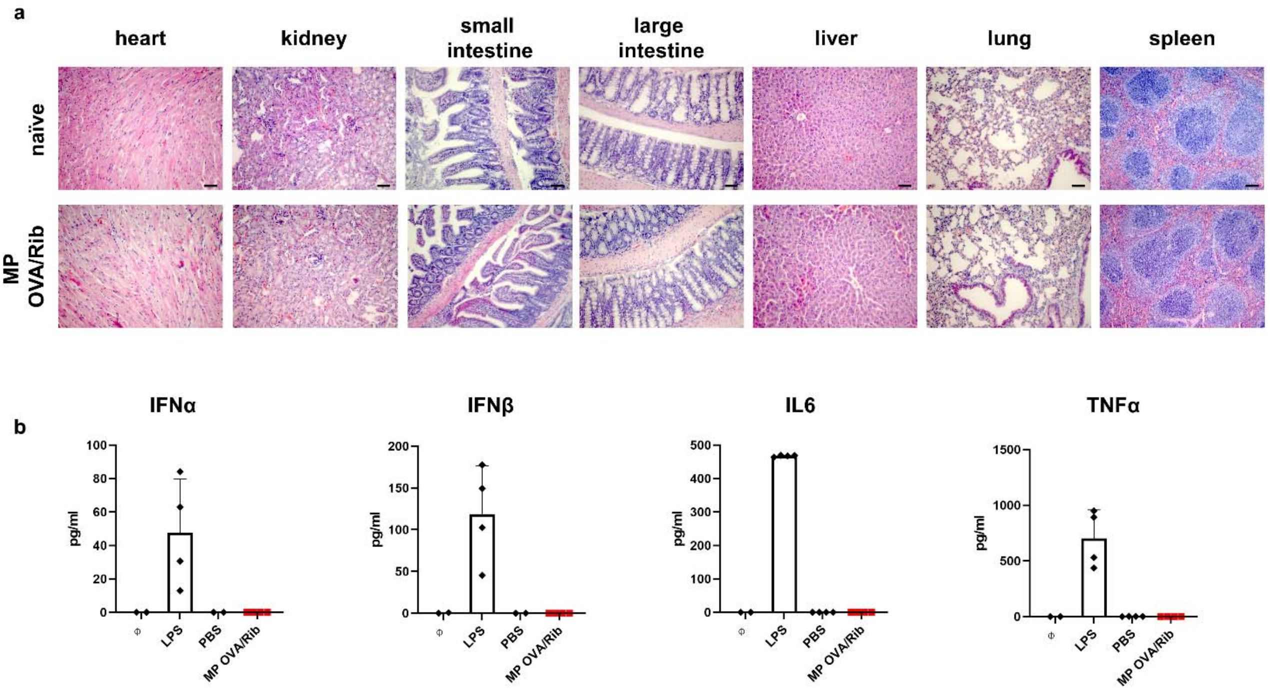

Supplementary Fig. 5│ Safety assessment of PLGA microparticle vaccination. a, C57BL/6J mice (n=5) were immunized s.c. with 5 mg microparticles (MP) loaded with 250 µg OVA and 2.5 µg Riboxxim (MP OVA/Rib, lower panel) in comparison to PBS treated control mice (naïve, upper panel). After 6 days, indicated organs were collected, paraffin-embedded and stained for H&E. Representative images (x200) are shown. Scale bars in top row represent 50 µm. Images in bottom row are of identical magnification. **b,** C57BL/6J mice (n=5) were immunized s.c. with 5 mg MP loaded with 250 µg OVA and 2.5 µg Riboxxim (MP OVA/Rib, red squares). Six days post immunization serum was collected and analyzed for type I interferons IFNα and IFNβ and for pro-inflammatory cytokine production (TNF, IL-6). Intraperitoneal injection of 10mg/kg LPS into C57BL/6 mice (black triangles, n=4) served as positive control for systemic cytokine secretion, whereas naïve C57BL/6 mice (black triangles, n=2) or C57BL/6 mice injected with 200µl PBS (black triangles, n=4) served as control mice. Data are presented as means ± SD.

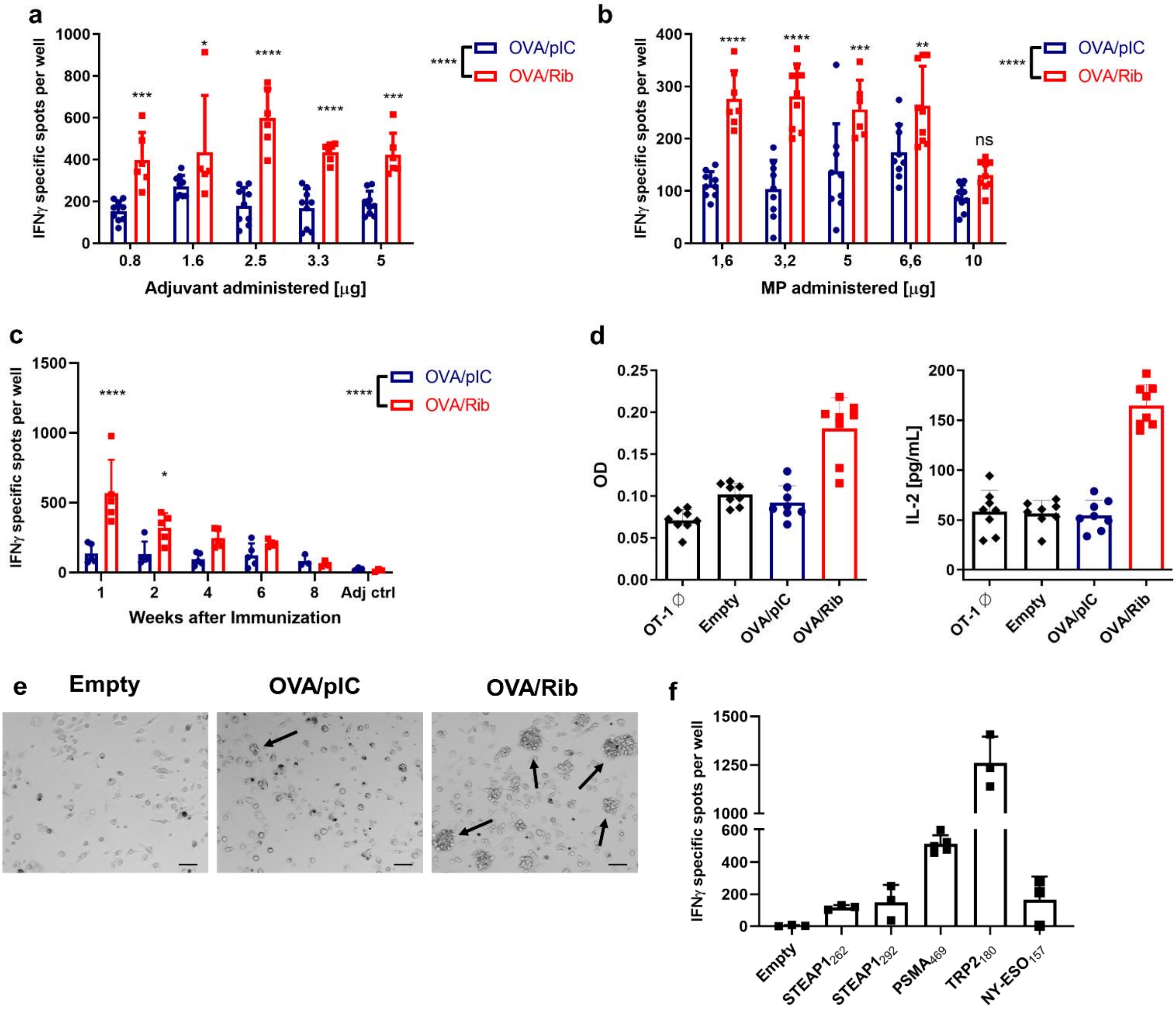

Supplementary Fig. 6 | Strength and duration of cytotoxic T cell response after vaccination of mice with PLGA microparticles. C57BL/6J mice were immunized s.c. with 5 mg of different MP charged with 250 µg OVA and 0.8 µg - 5 µg of polyI:C (pIC, blue circles, n=9) or Riboxxim (Rib, red squares, n=5-6) (**a**) or with 1.6 mg – 10 mg (**b**) of PLGA-MP-OVA/polyI:C (OVA/pIC, blue circles, n=9) or PLGA-MP-OVA/Riboxxim (OVA/Rib, red squares, n=6-9), respectively. After 6 days, splenocytes were isolated and analyzed for IFNγ production by ELISPOT assay. Background IFNγ spot formation (wells without peptide stimulation) was subtracted. The amount of IFNγ specific spots per well was analyzed by

automatic spot counting. Statistics: two-way ANOVA followed by Šídák's multiple comparisons test. *$P < 0.05$; **$P < 0.01$; ***$P < 0.001$; ****$P < 0.0001$; ns, not significant. **c**, Kinetics of the CTL response after a single immunization with MP-OVA/polyI:C or MP-OVA/Riboxxim. C57BL/6J mice (n=3-5) were immunized s.c. with 5 mg MP loaded with 250 µg OVA and 2.5 µg of dsRNA adjuvant (OVA/pIC, blue circles; OVA/Rib, red squares) or MP only carrying 2.5 µg dsRNA adjuvant as a control (Adj ctrl, n=2). IFNγ production of splenocytes was analyzed by ELISpot assay 6 days, 14 days, 28 days, 42 days and 56 days post immunization as described in (a,b). Statistics: two-way ANOVA followed by Šídák's multiple comparisons test. *$P < 0.05$; ****$P < 0.0001$. **d,** In vitro (cross-) presentation of PLGA-MP-OVA charged with polyI:C or Riboxxim. BMDCs (n=8) of naïve C57BL/6J mice were pulsed with 10 µg/mL MP-OVA/polyI:C (OVA/pIC, blue circles), MP-OVA/Riboxxim (OVA/Rib, red squares), or empty control MP (Empty, black diamonds) or left unpulsed (OT-1 Ø, black diamonds). After 24 hours, cells were incubated with either the OVA-specific $CD8^+$ T cell hybridoma B3Z (left panel) or with DOBW hybridoma cells (right panel) for 18 hours. Activation of B3Z was detected in a colorimetric *LacZ* assay and supernatants of DOBW hybridomas were analyzed for IL-2 production by ELISA. Summary of two experiments with means ± SEM is shown. **e,** Microscopic images of blasted OT-I cells (arrows) 24 hours after incubation with PLGA-MP pulsed BMDCs. Images are derived from two independent experiments with similar outcome. Microscopy images (x10) were acquired using AxioVision rel. 4.8.2 software equipped at the Axioplan 2 microscope (Carl Zeiss Imaging Inc.). Scale bars represent 50 µm. **f,** Tumor peptide-specific IFNγ production by splenocytes of AAD mice (n=3-5) immunized with PLGA-MP charged with 2.5 µg Riboxxim and 50 µg of indicated tumor peptide epitopes. ELISpot analyses was performed as described above. All data are presented as means ± SD of pooled data from at least two independent experiments with similar outcome.

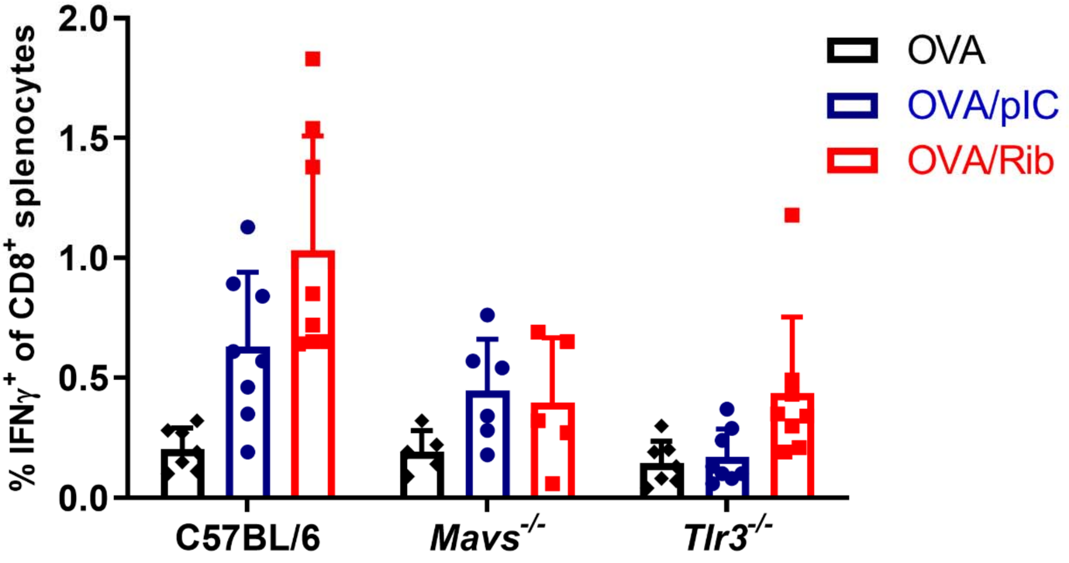

Supplementary Fig. 7│Analysis of cytotoxic T cell response after vaccination of WT, *Mavs*$^{-/-}$ or *Tlr3*$^{-/-}$ mice with PLGA microparticles. C57BL/6J mice (n=7-8), *Mavs*$^{-/-}$ mice (n=5-6) and *Tlr3*$^{-/-}$ mice (n=7-8) were immunized s.c. with 5 mg MP charged with 250 µg OVA and 2.5 µg dsRNA adjuvant (polyI:C, OVA/pIC, blue circles; Riboxxim, OVA/Rib, red squares). Six days post immunization, activation of antigen-specific cytotoxic T lymphocytes was monitored by intracellular cytokine staining for IFNγ^{+} of CD8^{+} splenocytes. Data are presented as means ± SD of pooled data from three independent experiments with similar outcome.

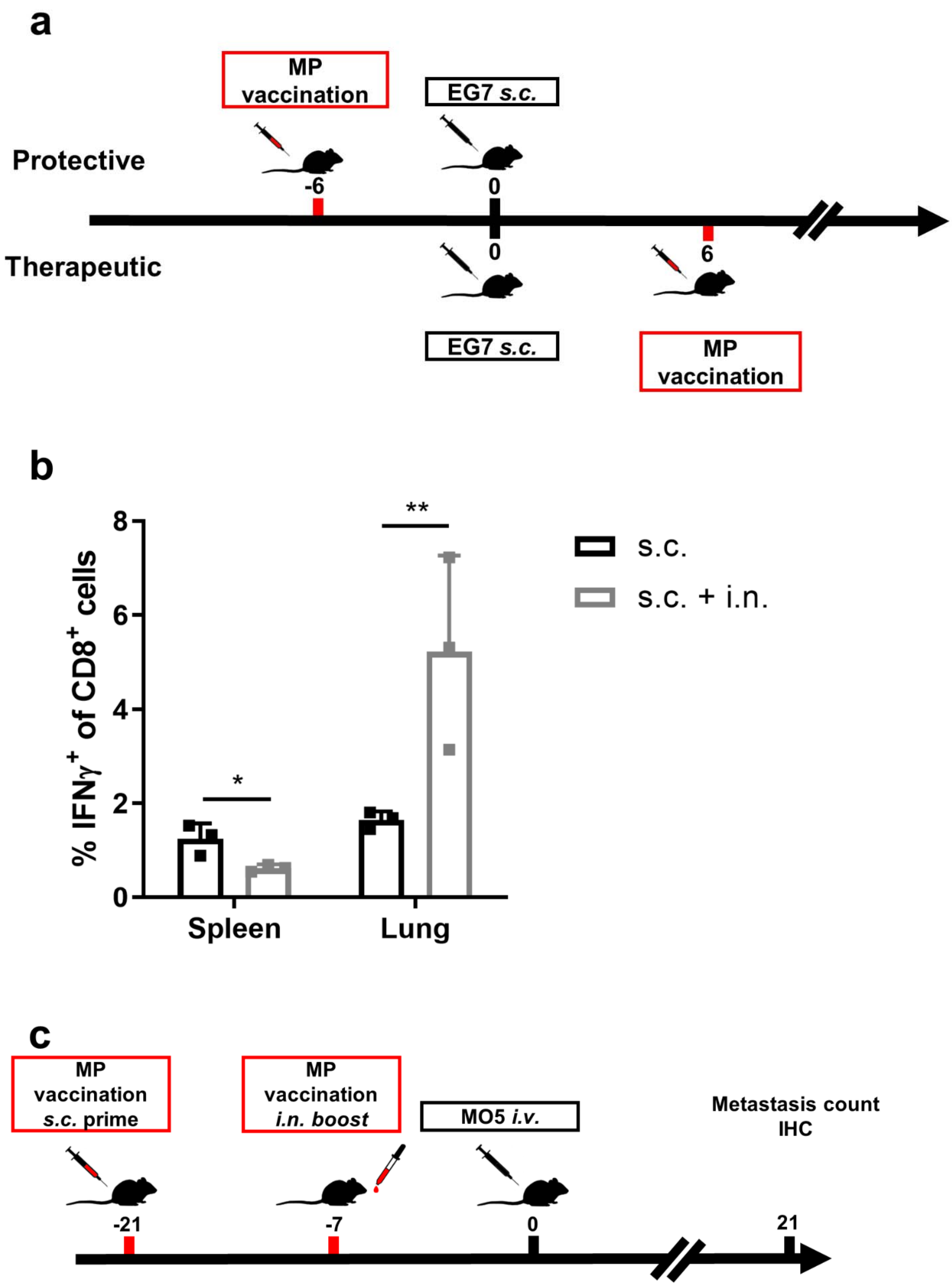

Supplementary Fig. 8│PLGA-microparticle (MP) vaccination directs anti-tumor response into the tumor microenvironment. a, Experimental scheme relevant to Fig. 5a,c. **b,**

Tumor-free C57BL/6J mice were either immunized with a single subcutaneous (s.c.) vaccination of PLGA-MP-OVA/Riboxxim (s.c., black squares) or received systemic subcutaneous prime and local intranasal (i.n.) boost application (s.c. + i.n., grey squares) of PLGA-MP-OVA/Riboxxim. 6 days after the last immunization, the percentage of IFNγ – positive $CD8^+$ cells in spleen and lung were analyzed by flow cytometry, indicating direct recruitment of tumor antigen-specific cytotoxic T lymphocytes into the tumor site. Data are presented as means ± SD. The experiment was repeated once with similar results. Statistics: two-way ANOVA with Šídák's multiple comparisons test. $^*P < 0.05$; $^{**}P < 0.01$. **c,** Experimental scheme relevant to Fig. 5e. Abbreviations: i.v., intravenous; IHC, immunohistochemistry.

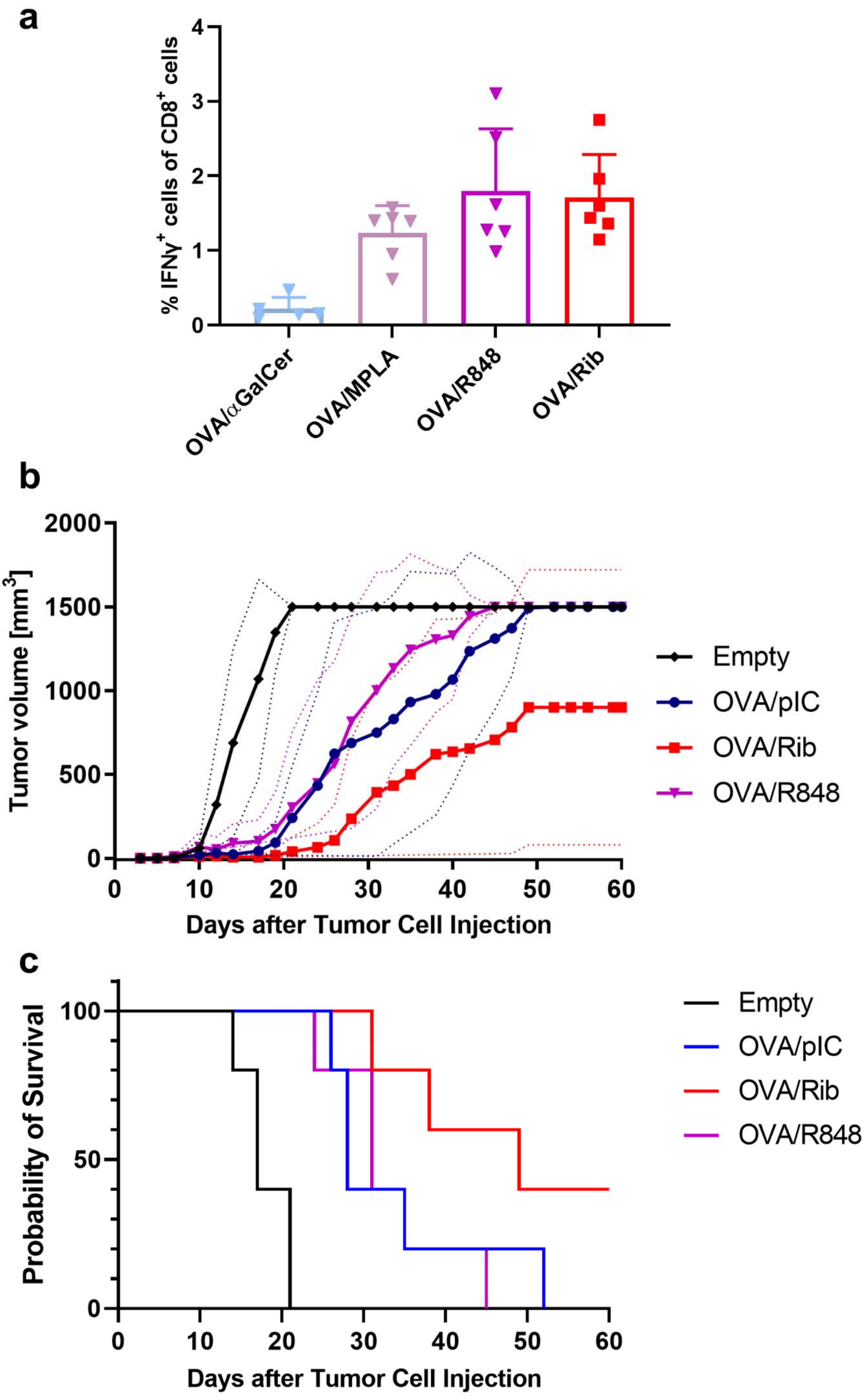
a
% IFNγ+ cells of CD8+ cells
4
3
2
1
0
OVA/αGalCer
OVA/MPLA
OVA/R848
OVA/Rib
b
Tumor volume [mm3]
2000
1500
1000
500
0
Days after Tumor Cell Injection
0
10
20
30
40
50
60
Empty
OVA/pIC
OVA/Rib
OVA/R848
c
Probability of Survival
100
50
0
Days after Tumor Cell Injection
Empty
OVA/pIC
OVA/Rib
OVA/R848

Supplementary Fig. 9│Comparative analysis of various PLGA-microparticle (MP) encapsulated adjuvants in T cell activation and antitumor efficacy. a, C57BL/6J mice were immunized subcutaneously (s.c.) with 5 mg MP charged with 250 µg OVA and 2.5 µg α-GalCer (OVA/αGalCer, blue triangles, n=5), MPLA (OVA/MPLA, lilac triangles, n=6), Resiquimod (OVA/R848, purple triangles, n=6) or Riboxxim (OVA/Rib, red squares, n=6). Six days post immunization, antigen-specific cytotoxic T lymphocyte activation was monitored by intracellular cytokine staining for $IFN\gamma^+$ of $CD8^+$ splenocytes. Data are presented as means ± SD representing pooled data derived from two independent experiments. **b,c,** C57BL/6J mice were s.c. challenged with $5x10^5$ E.G7-OVA-luc^+ cells. As soon as palpable tumors were detectable, mice were immunized with PLGA-MP-OVA/polyI:C (OVA/pic, blue circles, n=5), MP-OVA/Riboxxim (OVA/Rib, red squares, n=5), MP-OVA/Resiquimod (OVA/R848, purple triangles, n=5) or empty MP (Empty, black diamonds, n=5). Tumor growth curves (b) and Kaplan-Meier analysis of overall survival (c) are presented.

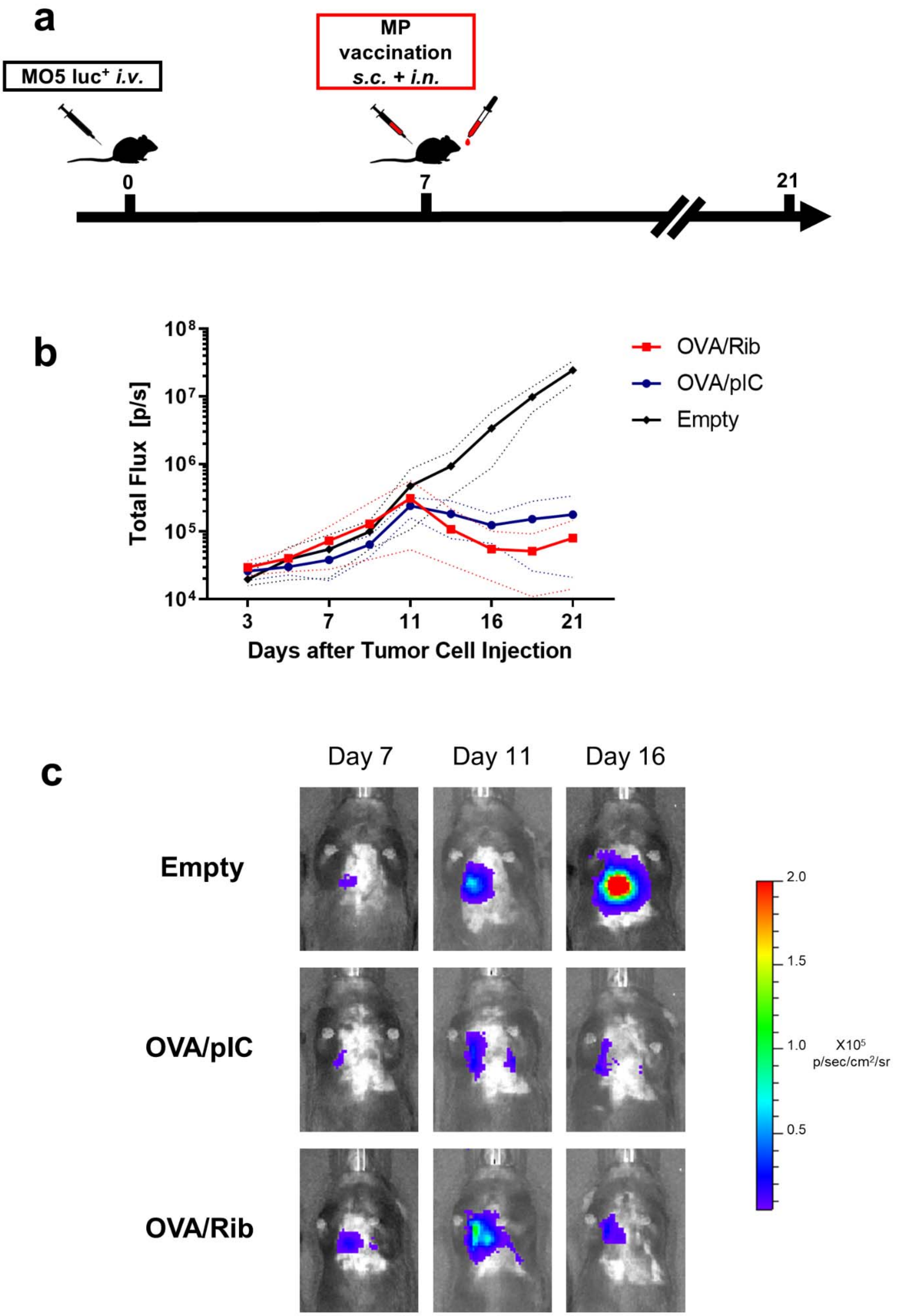

a
MO5 luc+ i.v.
MP vaccination s.c. + i.n.
0
7
21
b
Total Flux [p/s]
Days after Tumor Cell Injection
OVA/Rib
OVA/pIC
Empty
c
Day 7
Day 11
Day 16
Empty
OVA/pIC
OVA/Rib
X10^5 p/sec/cm^2/sr

Supplementary Fig. 10 | Antitumor efficacy of PLGA-MP containing tumor antigens and Riboxxim in a therapeutic lung metastasis model. a, Experimental setup of the therapeutic PLGA-MP treatment in the pulmonary metastasis model. C57BL/6J mice were injected intravenously (i.v.) with $1x10^5$ OVA/Luciferase-expressing MO5 melanoma cells (MO5-luc$^+$). As soon as bioluminescent signals were detectable in the lungs, mice were immunized by double-route immunization of s.c. administration of 5 mg MP charged with 250 µg OVA and 2.5 µg polyI:C (OVA/pIC, blue circles, n=5) or Riboxxim (OVA/Rib, red squares, n=5) and intranasal (i.n.) vaccination with 2.5 mg of the respective microparticles corresponding to 125 µg tumor antigen and 1.25 µg dsRNA adjuvants. Mice immunized with empty microparticles via the same regimen served as control (Empty, black diamonds, n=5). **b,** Tumor growth was quantitatively monitored over time by bioluminescence. Luminescent signal intensities were averaged from manually set ROI (regions of interest) and are presented as total flux (photons/seconds p/s). **c,** Representative IVIS® images on day 7, day 11 and day 16 after tumor cell inoculation demonstrating anti-tumor response and tumor regression after therapeutic PLGA-MP vaccination. Scaling of the pseudo color bioluminescent signals is depicted next to the luminescent images and presented as photons/seconds/cm^2/steradian (p/sec/cm/sr). The experiment was performed three times with similar outcome.

a

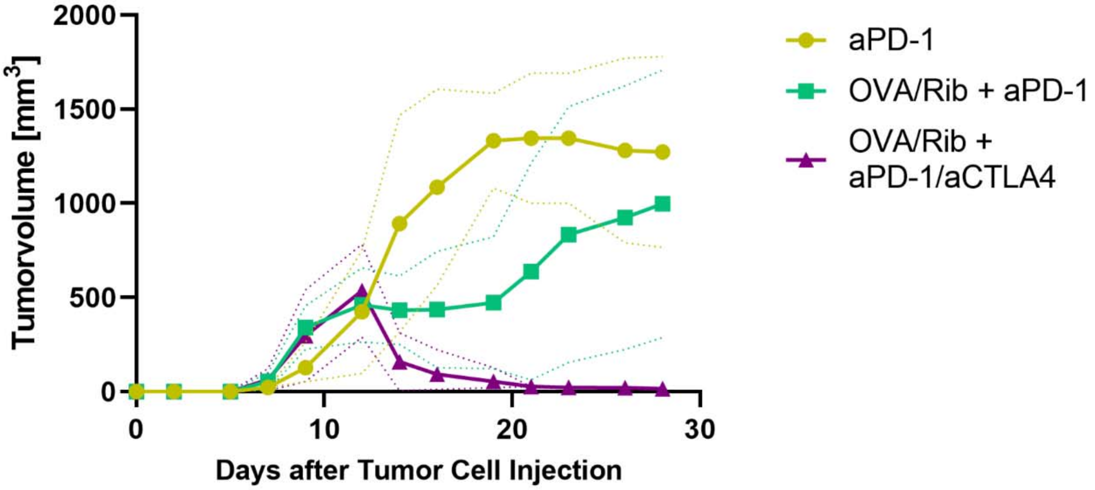

b

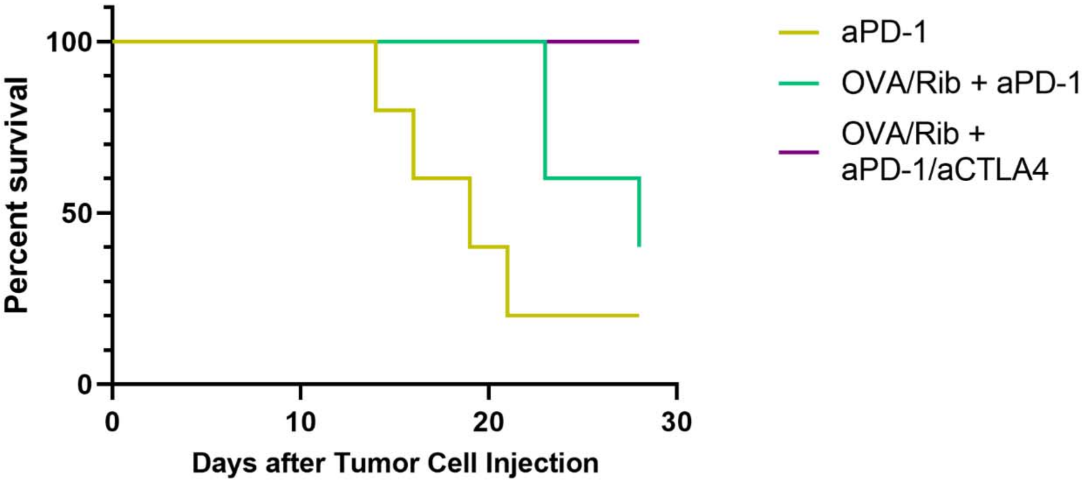

Supplementary Fig. 11│ Immune checkpoint blockade synergizes with therapeutic PLGA-MP cancer immunotherapy. a,b, E.G7-OVA-luc$^+$ tumors bearing C57BL/6J mice (n=5) were treated right after appearance of palpable tumors with PLGA-MP (250 µg OVA, 2.5µg Riboxxim) in combination with systemic anti-PD-1 (2000 µg/mouse) mAb (OVA/Rib + aPD-1, green squares, n=5) or with anti-CTL-4 (250 µg/mouse)/anti-PD-1 (2000µg/mouse) mAb (OVA/Rib + aPD-1/aCTLA4, purple triangles, n=5). As a control, C57BL/6 mice received anti-PD-1 (2000 µg/mouse) monotherapy (aPD-1, light green circles, n=5). Immune checkpoint

blockade was performed by i.p. injections of the antibodies on day 2, 4, 6 and day 8 post vaccination. Graphs with tumor growth curves (a) and Kaplan-Meier overall survival over time (b) are presented. Data were replicated in two independent experiments with similar outcome.

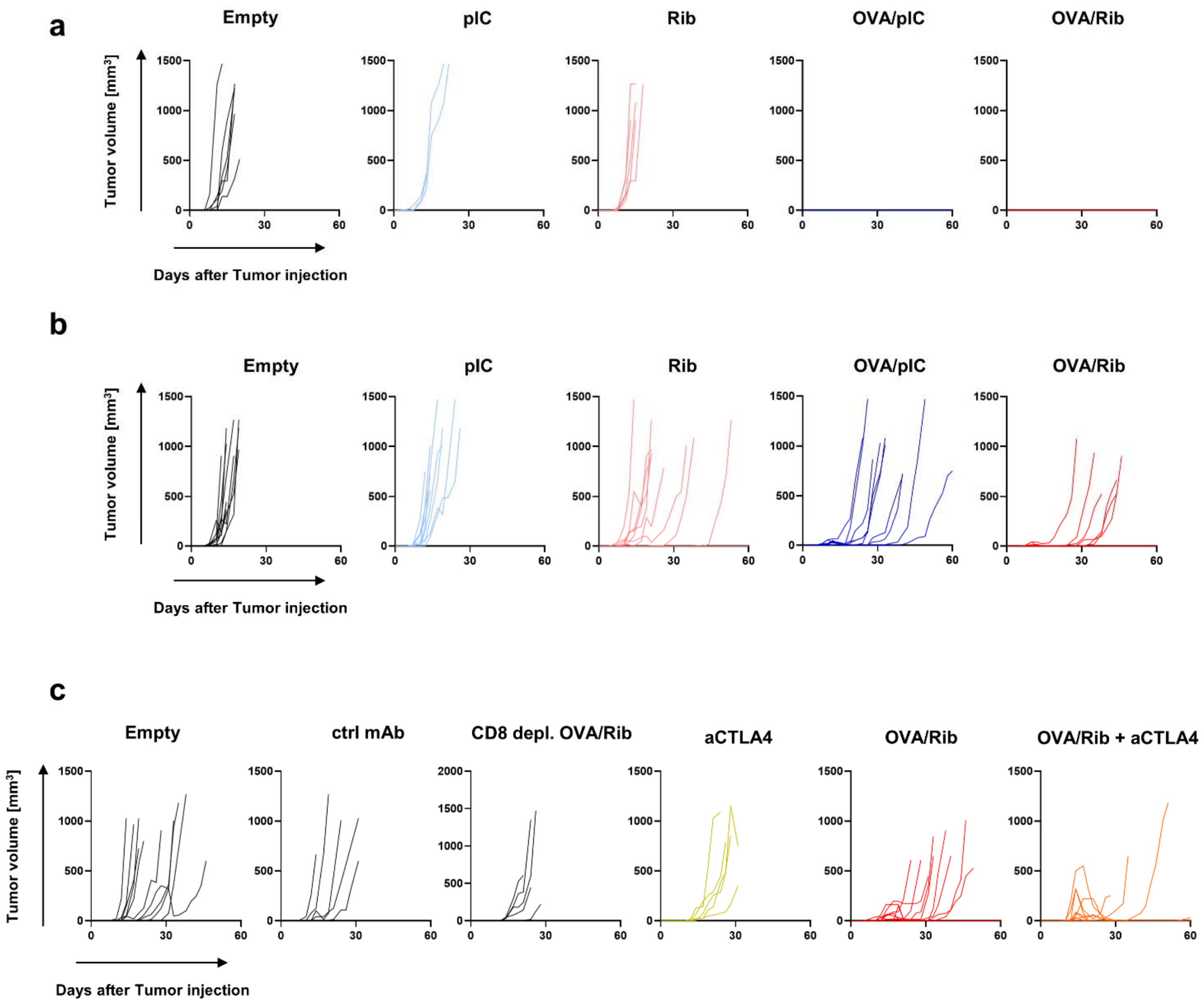

Supplementary Fig. 12 | Individual tumor growth data. a,b,c, Spider-plots of tumor growth of individual mice related to Figure 5a (a), Figure 5c (b) and Figure 6b (c).

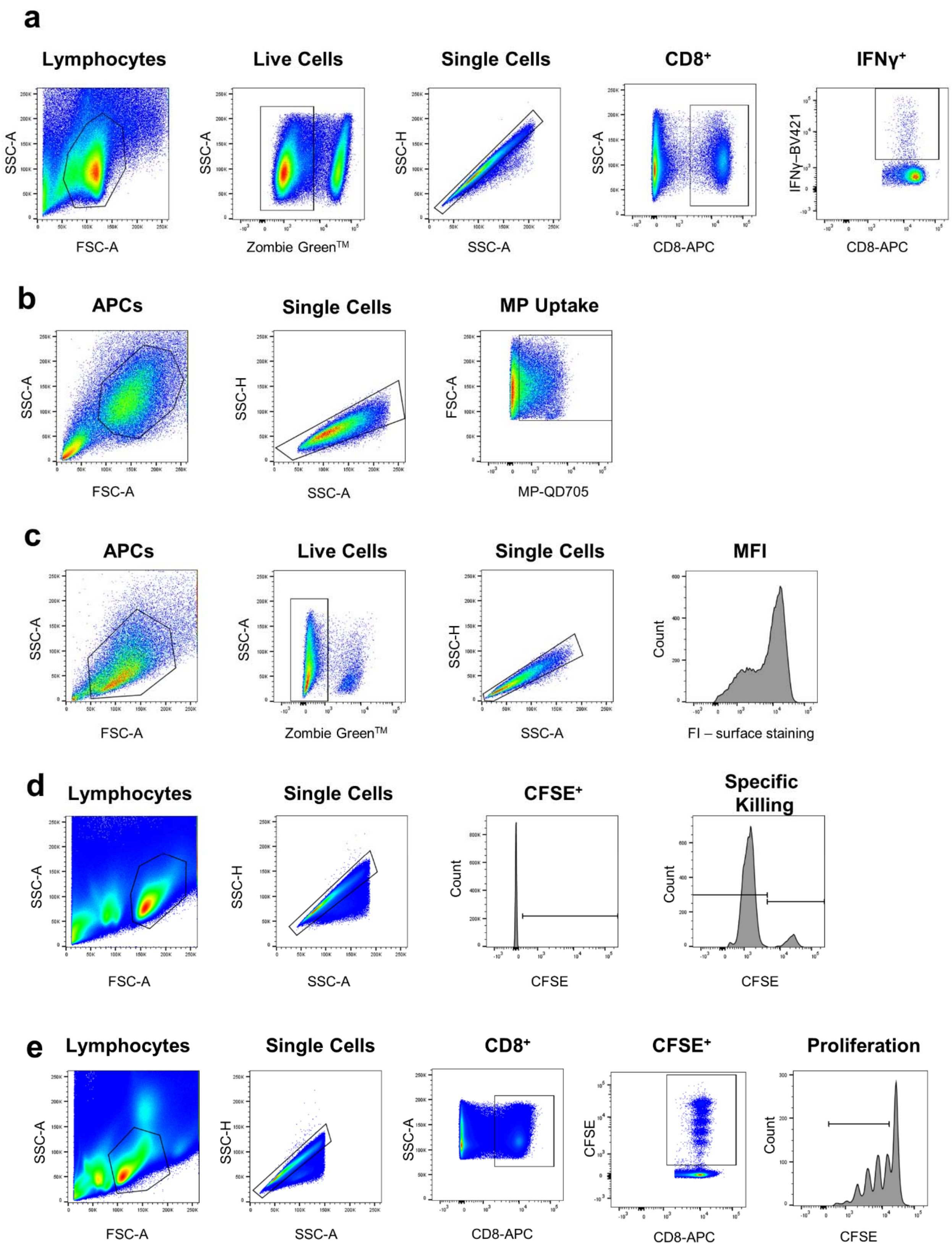

Supplementary Fig. 13│ Gating strategies used for flow cytometry. a, Gating strategy to determine the percentage of intracellular IFNγ of antigen-specific CD8+ T cells presented on

Fig. 1c,d, 4a,b,d,g, S1c,e, S7, S8b and Fig. S9a. Cells were gated using FSC/SSC followed by live/dead and singlet (single cells) discrimination with subsequent gating of $CD8^{+}$ and $IFN\gamma^{+}$ positive $CD8^{+}$ cells. **b,** Gating strategy to determine fluorescent particle uptake. Cells were gated using FSC/SSC to determine population of antigen-presenting cells (APC) followed by live/dead and singlet (single cells) discrimination with subsequent gating on MP-QD705 positive signals using the APC-R700 channel. Gating was applied in Fig. 2e and Fig. S2b. **c,** Gating strategy applied for analysis of activation and maturation marker on APCs after addition of soluble or particle bound dsRNA analogues. Cells were gated using FSC/SSC to determine APC population followed by live/dead and singlet discrimination with subsequent gating on positive signals of respective fluorochrome-labeled surface marker. Flow cytometric analysis was performed in Fig. 3a,c and Supplementary Figs. 3a,c,d, and 4b. **d,** Gating strategy to analyze antigen-specific killing of peptide-pulsed target cells in vivo. Cells were gated using FSC/SSC to specify lymphocytes followed by singlet discrimination and subsequent gating on CFSE positive signals. $CFSE^{+}$ signals were further gated on $CFSE^{low}$ and $CFSE^{high}$ populations to determine unpulsed reference cells and peptide-pulsed target cells, respectively. Results are shown in Fig. 4c. **e,** Gating strategy to determine in vitro proliferation of CD8+ T cells using proliferation and cell tracking dye. Cells were gated using FSC/SSC to specify lymphocytes followed by singlet discrimination and positive gating on CD8-positive cells and CFSE dilutions. Gating was applied in Fig. 4e.

Supplementary Table 1. List of primers for genotyping

Primer Name	Sequence (5'-3')
Mavs$^{-/-}$ mice	
Mavs$^{-/-}$ mutant	GTG GAA TGT GTG CGA GGC CAG AGG C
Mavs$^{-/-}$ wildtype	TAG CTG TGA GGC AGG ACA GGT AAG G
Mavs$^{-/-}$ common	AGC CAA GAT TCT AGA AGC TGA GAA
Tlr3$^{-/-}$ mice	
Tlr3$^{-/-}$ mutant	GCC AGA GGC CAC TTG TGT AG
Tlr3$^{-/-}$ wildtype	GCA ACC CTT TCA AAA ACC AG
Tlr3$^{-/-}$ common	AAT TCA TCA GTG CCA TGA GTT T
AAD mice	
H2D forward	ACG GAA AGT GAA GGC CCA CTC
H2D reverse	GCA GCC ATA CAT CCT CTG GAC G
Internal control forward	CAA ATG TTG CTT GTC TGG TG
Internal control reverse	GTC AGT CGA GTG CAC AGT TT'

Supplementary Table 2. List of antibodies for flow cytometry

Target	Clone	Specificity	Source	Catalog #	Used at
CD3ε	17A2	mouse	BioLegend	100204	1:200
CD8α	53-6.7	mouse	Invitrogen	17-0081-82	1:800
CD8α	53-6.7	mouse	BioLegend	100712	1:800
CD8α	53-6.7	mouse	BioLegend	100753	1:400
CD40	3/23	mouse	BioLegend	124612	1:200
CD80	16-10A1	mouse	BD Biosciences	553769	1:400
CD83	Michel-19	mouse	BD Biosciences	558206	1:400
CD86	GL1	mouse	BD Biosciences	560582	1:800
CD86	GL1	mouse	BioLegend	105012	1:800
H-2Kb	AF6-88.5	mouse	BioLegend	116508	1:600
H-2Kb	AF6-88.5	mouse	BioLegend	116518	1:600
IFNγ	XMG1.2	mouse	BioLegend	506538	1:1000
CD1c	L161	human	BioLegend	331526	1:50
CD1c (BDCA-1)	REA694	human	Miltenyi Biotec	130-110-594	1:20
CD141	1A4	human	BioLegend	565321	1:50
CD141 (BDCA-3)	REA674	human	Miltenyi Biotec	130-110-658	1:25
CD3ε	145-2C11	human	BioLegend	100306	1:50
CD14	M5E2	human	BioLegend	301804	1:50
CD16	B73.1	human	BioLegend	360716	1:50
CD19	HIB19	human	BioLegend	302206	1:50
CD68	Y1/82A	human	BioLegend	333806	1:50
CD40	HB14	human	BioLegend	313006	1:25
CD80	2D10.4	human	Invitrogen	17-0809-42	1:50
CD80	2D10	human	BioLegend	305222	1:50
CD80	L307	human	BD Biosciences	557227	1:20
CD83	HB15e	human	BD Biosciences	551073	1:25
CD83	HB15e	human	BioLegend	305312	1:50
CD83	HB15e	human	BioLegend	305324	1:100

CD86	BU63	human	BioLegend	374208	1:100
CD86	BU63	human	BioLegend	374212	1:100
CD86	BU63	human	BioLegend	374214	1:75
CD86	BU63	human	BioLegend	374206	1:50
HLA-A2	BB7.2	human	BioLegend	343307	1:100
HLA-A,B,C	W6/32	human	BioLegend	311432	1:150
HLA-A,B,C	W6/32	human	BioLegend	311425	1:150
