# Supplementary material for: PLGA-particle vaccine carrying TLR3/RIG-I ligand Riboxxim synergizes with immune checkpoint blockade for effective anti-cancer immunotherapy
Source: Nat Commun. 2021 May 18;12:2935. doi: 10.1038/s41467-021-23244-3 (PMC8131648; doi:10.1038/s41467-021-23244-3)
Supplement: Supplementary file 3 — Reporting summary [file 41467_2021_23244_MOESM3_ESM.pdf]

nature research

Corresponding author(s): Groettrup, Marcus

Last updated by author(s): Mar 22, 2021

Reporting Summary

Nature Research wishes to improve the reproducibility of the work that we publish. This form provides structure for consistency and transparency in reporting. For further information on Nature Research policies, seeAuthors & Referees and theEditorial Policy Checklist .

Statistics

For all statistical analyses, confirm that the following items are present in the figure legend, table legend, main text, or Methods section.

n/a	Confirmed	
☐	☒	The exact sample size (*n*) for each experimental group/condition, given as a discrete number and unit of measurement
☐	☒	A statement on whether measurements were taken from distinct samples or whether the same sample was measured repeatedly
☐	☒	The statistical test(s) used AND whether they are one- or two-sided<br>*Only common tests should be described solely by name; describe more complex techniques in the Methods section.*
☒	☐	A description of all covariates tested
☒	☐	A description of any assumptions or corrections, such as tests of normality and adjustment for multiple comparisons
☐	☒	A full description of the statistical parameters including central tendency (e.g. means) or other basic estimates (e.g. regression coefficient) AND variation (e.g. standard deviation) or associated estimates of uncertainty (e.g. confidence intervals)
☐	☒	For null hypothesis testing, the test statistic (e.g. *F*, *t*, *r*) with confidence intervals, effect sizes, degrees of freedom and *P* value noted<br>*Give P values as exact values whenever suitable.*
☒	☐	For Bayesian analysis, information on the choice of priors and Markov chain Monte Carlo settings
☒	☐	For hierarchical and complex designs, identification of the appropriate level for tests and full reporting of outcomes
☒	☐	Estimates of effect sizes (e.g. Cohen's *d*, Pearson's *r*), indicating how they were calculated

Our web collection on statistics for biologists contains articles on many of the points above.

Software and code

Policy information about availability of computer code

Data collection	All flow cytometric data were collected using FACSuite™ v1.2.1 software at the BD FACSLyric™ flow cytometer and FACSDiva™ v8.0.1 at the BD LSRFortessa™ (all BD Biosciences) . Scanning electron microscopy (SEM) images were acquired using SmartSEM® v6 software at the Auriga® scanning electron microscope. ImmunoSpot® Smart Count™ v.6.0.0.2 was used for ELISpot automated spot counting. Bioluminescence and Fluorescence Imaging was performed using Living Image 4.1. Magellan™ Software was utilized for all spectrophotometric measurements using Tecan™ microplate reader. ZEN 3.2 (blue edition) imaging software was applied for acquisition of immunohistochemical images. Microscopy images were acquired using AxioVision (Rel. 4.8) software equipped at the Axioplan 2 microscope (Zeiss). Particle size and surface charge was measured using Zetasizer Software Version 7.12 (Malvern Pananalytical) at the Zetasizer ZSP Nano device.
Data analysis	Source data were processed and statistical analysis was performed using GraphPad Prism 8.4 (GraphPad Software, Inc.). All flow cytometry data were analyzed using FlowJo software V10.1 (BD Biosciences). (GraphPad Software, Inc.). ImageJ (FIJI) v1.53c was used as image processing software.

For manuscripts utilizing custom algorithms or software that are central to the research but not yet described in published literature, software must be made available to editors/reviewers. We strongly encourage code deposition in a community repository (e.g. GitHub). See the Nature Research guidelines for submitting code & software for further information.

Data

Policy information about availability of data

All manuscripts must include a data availability statement. This statement should provide the following information, where applicable:

- Accession codes, unique identifiers, or web links for publicly available datasets
- A list of figures that have associated raw data
- A description of any restrictions on data availability

The authors declare that all source data (graphs) for the main figures of this study are available within the manuscript and within the Supplementary Information. The source data are provided as a Source Data File within the article. A data availability statement is included in the manuscript.

Additional data and source files are available from the corresponding author upon reasonable request.

Field-specific reporting

Please select the one below that is the best fit for your research. If you are not sure, read the appropriate sections before making your selection.

- [x] Life sciences
- [ ] Behavioural & social sciences
- [ ] Ecological, evolutionary & environmental sciences

For a reference copy of the document with all sections, see nature.com/documents/nr-reporting-summary-flat.pdf

Life sciences study design

All studies must disclose on these points even when the disclosure is negative.

Sample size

For animal studies, the sample size was defined on the basis of similar experiments either from previous studies cited in the manuscript or taken from literature. Group sizes were determined using G*Power (Version 3.1.9.2) by providing an effect of 20% difference between the groups with a significance level of 5% and statistical power of 95%. For ethical reasons, the minimum number of animals necessary to achieve the calculated statistical significances was used. For experiments other than animal studies, a minimum of triplicates was chosen to allow for calculation of statistics. All results have been independently reproduced at least once. The sample size for individual data was determined according to previous experiments of similar type by taking in consideration the means of target values between the experimental group and the control group and the statistical analysis used in order to obtain statistical differences.

Data exclusions

No data were excluded from the analysis. Data concerning human myleoid DCs CD1+ and CD141+ had to be performed with only low numbers of cells due to the low abundance of these cells in human PBMC fractions.

Replication

In vivo studies were performed using at least 3 animals per group. Sample sizes are stated in the figure legends. Data are hereby presented as means ± SD of at least two independent replicates of the same experiment. Some tumor experiment were only repeated once with similar succesful outcome. All othe data are also presented as mean ± SD of at least 3 independent replicates of the same experiment with similar results - if not otherwise stated in the figure legends. Microscopy images (SEM, IHC, H&E, proliferation) were generated and collected of representative data or individual animals from at least 2 independent experiments using at least 3 different replicates. Similar results has been obtained from these independent experiments.

Randomization

Experimental animals were randomly distributed to different treatment groups. Concerning tumor experiments, animals were randomly distributed into control and experimental groups in the protective tumor setting, as well as after tumor cell inoculation in therapeutic tumor settings. For experiments other than mice studies, samples were also randomly allocated into experimental groups. Often, treatment groups have to be applied to the same experimental source (e.g. BMDCs). Randomization of the experimental groups was applied by altering location of treatment groups in the plate between different experiments to ensure that location had no effect on the results.

Blinding

Immunohistochemical analysis of tumor sections and major organs was performed in a double-blinded manner. As stated above, animals were randomly divided into control and experimental groups. However, immunizations and antibody injections were performed using established treatment schedules and treatment regimens and could not be performed blinded. Data acquisition was inherently blinded since measurements were performed by the respective instruments in automated, unbiased manner.

Reporting for specific materials, systems and methods

We require information from authors about some types of materials, experimental systems and methods used in many studies. Here, indicate whether each material, system or method listed is relevant to your study. If you are not sure if a list item applies to your research, read the appropriate section before selecting a response.

Materials & experimental systems

n/a	Involved in the study	
[ ]	[x]	Antibodies
[ ]	[x]	Eukaryotic cell lines
[x]	[ ]	Palaeontology
[ ]	[x]	Animals and other organisms
[ ]	[x]	Human research participants
[x]	[ ]	Clinical data

Methods

n/a	Involved in the study	
[x]	[ ]	ChIP-seq
[ ]	[x]	Flow cytometry
[x]	[ ]	MRI-based neuroimaging

Antibodies

Antibodies used

Antibodies for flow cytometry are additionally listed in the Supplementary Informations of this article as supplementary Table II with description of used dilutions.

FLOW CYTOMETRY:
anti-mouse primary antibodies:

CD3-FITC (Biolegend, #100204, Lot: B275312, clone: 17A2)
CD8a-APC (Invitrogen, Cat.#17-0081-82, Lot: 195304, clone: 53-6.7)
CD8a-APC (Biolegend, #100712, Lot:B288881, clone: 53-6.7)
CD8a-BV421 (Biolegend, #100753, Lot: B263165, clone: 53-6.7)
IFNg-BV421 (Biolegend, #506538, Lot: B523728, clone: XMG1.2)
CD40-APC (Biolegend, #124612, Lot: B248272, clone: 3/23)
CD80-PE (BD Biosciences, #553769, Lot: 6019887, Lot: 28825, clone: 16-10A1)
CD83-APC (BD Biosciences, #558206, Lot: 7118711, clone: Michel-19)
CD86-PE (BD Biosciences, #560582, Lot: 4073721, Lot: 6134585, clone: GL1)
CD86-APC (Biolegend, #105012, Lot: B245161, clone: GL-1)
H-2Kb-PE (Biolegend, #116508, Lot: B238421, clone: AF6-88.5)
H-2Kb-APC (Biolegend, #116518, Lot: B249127, clone: AF6-88.5)

anti-human primary antibodies:
CD1c-BV421 (Biolegend, #331526, Lot: B201898, clone: L161)
CD1c (BDCA-1)-PE (Miltenyi-Biotec, #130-110-594, Lot: 5190613236, clone: REA694)
CD141-BV421 (BD Biosciences, #565321, Lot: 9322743 , clone: 1A4)
CD141 (BDCA-3)-APC (Miltenyi Biotec, #130-110-658, Lot: 5190829130, clone: REA674)
CD3e-FITC (Biolegend, #100306, Lot: B218086 clone: 145-2C11)
CD14-FITC (Biolegend, #301804, Lot: B268130 , clone: M5E2)
CD16-FITC (Biolegend, #360716, clone: B73.1)
CD19-FITC (Biolegend, #302206, Lot: 39447, clone: HIB19)
CD68-FITC (Biolegend, #333806, clone: Y1/82A)
CD40-PE (Biolegend, #313006, Lot: B257400, clone: HB14)
CD80-APC (Invitrogen, #17-0809-42, Lot: 2103129, clone: 2D10.4)
CD80-BV421 (Biolegend, #305222, Lot: B255304, clone: 2D10)
CD80-PE (BD Biosciences, # 557227, Lot: 5093990, clone: L307)
CD83-APC (BD Biosciences, #551073, Lot:36256, clone: HB15e)
CD83-APC (Biolegend, #305312, Lot: B205335, clone: HB15e)
CD83-BV421 (Biolegend, #305324, Lot: B261466, clone: HB15e)
CD86-APC (Biolegend, #374208, Lot: B262802, clone: BU63)
CD86-BV421 (Biolegend, #374212, Lot: B262217, clone: BU63)
CD86-BV605 (Biolegend, #374214, Lot: B288319, clone: BU63)
CD86-PE (Biolegend, #374206, Lot: B246947, clone: BU63)
HLA-A2-APC (Biolegend, #343307, Lot: B254384, clone BB7.2)
HLA-A,B,C-BV605 (Biolegend, #311432, Lot: B277485, clone: W6/32)
HLA-A,B,C-APC-Cy7 (Biolegend, #311425, Lot: B267287, clone: W6/32)

Zombie Green™ Fixable Viability Kit (Biolegend, #423112, Lot: B259362, dilutions: 1:100-1:1000)
CFSE (Biolegend, #423801, Lot: B270980)
Purified Rat Anti-Mouse CD16/CD32 (Mouse BD Fc-Block™ (BD Biosciences, #553141, Lot:5154726, clone: 2.4G2)

GoInVivo™ Purified anti-mouse CD152 Antibody (anti-CTLA-4) (Biolegend, #106205, Lot: B227309, clone: 9H10)
GoInVivo™ Purified Syrian Hamster IgG Isotype Ctrl Antibody (Biolegend, #402019, Lot: B233734, clone: SHG-1)
InVivoMAb anti-mouse CD8β (Lyt 2.1) mAb (BioXCell, #BE0118, clone: HB129/116-13.1)
anti-PD-1 mAb (provided by Prof. H. Yagita, clone: RMPI-14)

HISTOLOGY:
CD8a (D4W2Z) XP® Rabbit mAb (mouse specific) (CST, #98941 , Lot: 2-12/2008, dilution: 1:200)

ELISPOT ASSAY:
ELISPOT-Pair #551881 (Capture Antibody, Lot: 7353898, dilution: 1:400; Detection Antibody, Lot: 7353886, dilution: 1:400) (BD Biosciences,)
Elispot HRP Streptavidin (BD Biosciences, #557630, Lot: 8123790, dilution: 1:1000)

ELISA-KITS:
Verikine™ Mouse Interferon Alpha ELISA Kits (PBL Assay Science, #42120-1, Lot: 6827, Lot: 7004; Lot; 6877)
LEGEND MAX™ Mouse IFN-ß ELISA Kit (Biolegend, #439408, Lot: B273501)
Mouse TNF alpha ELISA Ready-SET-Go! (affymetrix, eBioscience, #88-7324-88, Lot: E09483-1667)
Mouse IL-1 beta ELISA Ready-SET-Go! (affymetrix, eBioscience, #88-7013-88, Lot: E09327-1645)
Mouse IL-6 Uncoated ELISA (Invitrogen, #88-7064)
Mouse IL-12 p70 ELISA Ready-SET-Go! (affymetrix, eBioscience, #88-7121-88, Lot: E09401-1643)
Verikine™ Human Interferon Alpha All Subtype ELISA Kits (PBL Assay Science, #41115-1)
Verikine™ Human Interferon Beta ELISA Kits (PBL Assay Science, #41410-1)
Human TNF alpha Uncoated ELISA Kit (Invitrogen, #88-7346-88)

Human IL-1 Uncoated ELISA Kit (Invitrogen,, #88-7261-88)
Human IL-6 Uncoated ELISA Kit (Invitrogen, #88-7066-88)
Human IL-12 p70 Uncoated ELISA Kit (Invitrogen, #88-7126-88)

CYTOKINES:
recombinant human GM-CSF (Peprotech, # 300-03, Lot: 011830)
recombinant human IL-4 (Peprotech, #200-04, Lot: 041814)
recombinant murine GM-CSF (Peprotech, #315-03, Lot: 011655-1)

TLR LIGANDS/ADJUVANTS:
poly(I:C) HMW VacciGrade (TM) (Invivogen, #vac-pic, Lot: VPIC-38-01)
polyI:C sodium salt (Merck, #P0913, Lot: 095M4050V)
Riboxxim GMP certified (Riboxx, Lot: P04-ENQ-039-210317)
MPLA-SM VacciGrade™, (Invivogen, #vac-mpla)
R848 VacciGrade™ (Invivogen, #vac-r848)
α-Galactosylceramide (Funakoshi, #KRN7000)

D-Luciferin*K (Iris Biotech, #LS-1206.1000, Lot: 022726)
D-Luciferin potassium salt (Synchem, #bc219, Lot: XXXX)

CELL ISOLATION KITS/MICROBEADS:
CD1c (BDCA-1)+ Dendritic Cell Isolation Kit, human (Miltenyi Biotec, #130-119-475, Lot: 5191022244)
CD14 MicroBeads, human (Miltenyi Biotec, #130-050-201, Lot: 5181102249)
CD141 (BDCA-3) MicroBead Kit, human (Miltenyi-Biotec, #130-090-512, Lot:5190924692)
CD8a (Ly-2) MicroBeads, mouse (Miltenyi Biotec, #130-117-044, Lot: 5190813366)
Lung Dissociation Kit, mouse (Miltenyi Biotec, #130-095-927, Lot:5200910835)

ToxinSensor™ Chromogenic LAL Endotoxin Assay Kit (GenScript, #L00350C, Lot: C50181710)

Resomer RG502H (Evonik, #99023760; Lot: D180900529
Poloxamer 188 Solution (Merck, #P5556, Lot: RNBG9031)

Deep Blue Cell Viability™ (Biolegend, #424701, Lot: B297265)

Albumin from chicken egg white (Merck, #A5503, Lot: SLBQ9036V-10M; #A251, Lot: SLBT1992-6M)

Validation

All primary antibodies used for flow cytometry were validated by the manufacturer. Methods for validation are specifically supplied by the manufacturer's homepages. By entering provided catalog or lot numbers of primary antibodies at the provided links below, respective data sheets, certificate of analysis and specific information about validation of the antibodies are available.

Biolegend: https://www.biolegend.com/en-us/reproducibility

Invitrogen/eBioscience/affymetrix: https://www.thermofisher.com/de/de/home/life-science/antibodies/invitrogen-antibody-validation.html#:~:text=%20Invitrogen%20Antibody%20Validation*%20%201%20Antibody%20validation,for%20set%20up,%20designing,%20doing%20and...%20More

BD Biosciences: https://regdocs.bd.com/regdocs/qcinfo

Miltenyi-Biotec: https://www.miltenyibiotec.com/DE-en/resources/technical-documents/certificates.html

Cell Signaling (CST): https://www.cellsignal.de/about-us/cst-antibody-validation-principles

BioXcell: https://bxcell.com/the-bio-x-cell-advantage/

Antibody dye titration was performed before the according experiment based on the same specimen to determine the optimal dilution factor.

For IHC, staining was performed on 3 µm formalin-fixed paraffin embedded (FFPE) tissue sections. Optimal antibody concentration was determined before staining on experimental sections by antibody dilution series using positive control tissue sections. 3,3-diaminobenzidine tetrahydroxychloride (DAB) was used as chromogen and the slides were counterstained with hematoxylin. Control stainings were performed without addition of the primary antibody.

The specificity of the anti-PD-1 antibody was validated by FACS staining of mouse PD-1 transfectants as reported in the original paper for RMP1-14 (Yamazaki T. et al: Blockade of B7-H1 on macrophages suppresses CD4+ T cell proliferation by augmenting IFN-gamma-induced nitric oxide production. J Immunol. 2005 Aug 1;175(3):1586-92. doi: 10.4049/jimmunol.175.3.1586. PMID: 16034097.)

Eukaryotic cell lines

Policy information about cell lines

Cell line source(s)

The murine lymphoma cell line E.G7-OVA-luc+ (originally ATCC® CRL-2113™)was received from Thomas Blankenstein (MDC Berlin, Germany).
The murine melanoma tumor cell line MO5 was provided by Antje Heit (TU Munich, Germany).
The murine melanoma tumor cell line MO5-luc+ was generated by Anna MacKerracher (University of Konstanz, Germany; the original cell line B16BL6-luc+/GFP+ was kindly provided by Prof. Olaf van Tellingen (The Netherlands Cancer Institute, Amsterdam, The Netherlands))
The murine immature dendritic cell line DC2.4 (#SCC142, Merck) was gifted by Kenneth Rock (UMass Medical School, Worcester, MA, USA).
The murine CD8+ splenic DC cell line MutuDC2114 was obtained from Hans Acha-Orbea (University of Lausanne, Switzerland).
The human monocytic cell line THP-1 (ATCC® TIB-202™) was obtained from American Type Culture Collection (ATCC, Manassas, VA, USA).
The B3Z CD8+ T-cell hybridoma was a kind gift from Prof. Nilabh Shastri (University of California, Berkeley, USA).
The CD4+ T-cell hybridoma DOBW was kindly contributed by Prof. Clifford V. Harding (Washington University, School of Medicine, St. Louis, USA).

Authentication

No authentication method was performed by cell lines received from ATCC. Cell lines donated from other research group were authenticed at corresponding labs. We further authenticied cell lines by morphology according to data sheets provided at ATCC or similar webpages. Luciferase expression was tested and validated before injection of tumor cells into mice by in vitro bioluminescence assay. Expression of ovalbumin was authenticated via immunoblotting using anti-chicken egg albumin antibody (#C6534, Merck).

Mycoplasma contamination

Cell lines are regularily tested for mycoplasma contamination in cell culture supernatants at Microsynth, Switzerland. All used cell lines were negative for mycoplasma.

Commonly misidentified lines
(See ICLAC register)

No commonly misidentified cell lines were used.

Animals and other organisms

Policy information about studies involving animals; ARRIVE guidelines recommended for reporting animal research

Laboratory animals	-male and female C57BL/6J mice<br>-female BALB/cAnNCrl (Balb/c)<br>-female C57BL/6-Tg(TcraTcrb)1100Mjb/J (OT-1) mice<br>-male and female B6;129-Mavstm1Zjc/J (Mavs-/-) mice<br>-male and female B6;129S1-Tlr3tm1Flv/J (Tlr3-/-)<br>-female and male B6.Cg-Immp2lTg(HLA-A/H2-D)2Enge/J (AAD) mice. AAD mice were crossed with C57BL/6J mice. AAD-transgene detection was performed using following primers: H2D forward (5'-ACG GAA AGT GAA GGC CCA CTC-3') and H2D reverse (5'-GCA GCC ATA CAT CCT CTG GAC G-3'); PCR was controlled by following internal primer: control forward (5' CAA ATG TTG CTT GTC TGG TG') and control reverse (5'GTC AGT CGA GTG CAC AGT TT'). PCR product of AAD was at 350 bp, while internal control bands run at 200 bp.<br><br>Experimental animals were used at 8 to 12 weeks of age.
Wild animals	No wild animals were used in this study
Field-collected samples	No filed-collected samples were used in this study.
Ethics oversight	Animal experimentation were performed in compliance with ethical standards of German and EU guidelines after approval by the animal experimentation ethics committee of the Review Board of Governmental Presidium Freiburg, Germany. Several approvals were used for this study: G-15/102, G-16/81, G-19/176, G-20/03, G-20/143.

Note that full information on the approval of the study protocol must also be provided in the manuscript.

Human research participants

Policy information about studies involving human research participants

Population characteristics	Human PBMCs were collected by healthy volunteers of different age and sex and without any clinical manifestations.
Recruitment	Recruitment was performed by public postings at the University of Konstanz. No self-selection bias or other bias was applied.
Ethics oversight	Human specimens were used in accordance with the requirements of the institutional ethics committee and national policies. Blood donations were approved by the Ethics Committee of the University of Konstanz, Germany. Written informed consent was obtained from each randomly enrolled healthy volunteers before blood donation following guidelines of the Review Board protocol of the University of Konstanz. Blood donations were performed by the transfusion unit at the Klinikum Konstanz in accordance with the precepts of the transfusion unit at the hospital and of the DRK (German Red Cross) with all applicable regulations, guidance and local medical policies.

Note that full information on the approval of the study protocol must also be provided in the manuscript.

Flow Cytometry

Plots

Confirm that:

- [x] The axis labels state the marker and fluorochrome used (e.g. CD4-FITC).
- [x] The axis scales are clearly visible. Include numbers along axes only for bottom left plot of group (a 'group' is an analysis of identical markers).
- [x] All plots are contour plots with outliers or pseudocolor plots.
- [x] A numerical value for number of cells or percentage (with statistics) is provided.

Methodology

Sample preparation	Single cell suspensions of spleen and lymph nodes were obtained by mechanical disruption of the target tissues through 70 µm nylon mesh filters (BD Falcon) to remove debris. Obtained single-cell suspension were further cultivated in vitro or incubated with re-stimulating peptides for intracellular cytokine stainings or directly stained with flourochrome-labeled antibodies for surface markers. If needed, red blood cell (RBC) lysis was performed using RBC-Lysis Buffer (NH4Cl, KHCO3, EDTA). Lung tissue was dissociated using the mouse Lung Dissociation Kit (#130-095-927, Miltenyi Biotec) with a gentleMACS™ octo Dissociater according to the manufacturer's protocol. Bone-marrow derived dendritic cells were isolated from femur and tibia of naive mice according to established protocols by Lutz, M. B. et al. An advanced culture method for generating large quantities of highly pure dendritic cells from mouse bone marrow. J. Immunol. Methods 223, 77–92 (1999). Human peripheral blood mononuclear cells (PBMC) were isolated by Ficoll Paque (GE healthcare) density centrifugation from whole blood donated by healthy volunteers. If needed, target cell separation was performed using MACS® MicroBeads technology according to the manufacturer's protocol (Miltenyi Biotec) yielding almost pure cell populations for further use.

Instrument	BD FACSLyric™ and BD LSRFortessa™ (BD Biosciences)
Software	Data collection was performed by FACSuite™ v1.2.1 software at the BD FACSLyric™ flow cytometer and FACSDiva™ v8.0.1 at the BD LSRFortessa™ (all BD Biosciences). Data were analyzed using FlowJo software V10.1 (TreeStar, Ashland, OR).
Cell population abundance	no FACS sorting was performed during this study
Gating strategy	Gating strategies relied on preliminary identification of lymphocyte or monocyte gates in FSC/SSC scatter plots, followed by doublet exclusion and positive gating on live, single cells after dead cell exclusion. Positive signals were further identified by double positive staining for intracellular cytokines or positive gating on target cell populations and analysis of surface marker. Especially for human myleoid cells, cells were identified by exclusion of other Lineage markers in the FITC-channel. Remaining cells were identified by positive gating on CDc1+ or CD141+ cells and further analysis of surface markers.

☒ Tick this box to confirm that a figure exemplifying the gating strategy is provided in the Supplementary Information.
